# How C-Reactive Protein Structural Isoforms With Distinctive Bioactivities Affect Disease Progression

**DOI:** 10.3389/fimmu.2020.02126

**Published:** 2020-09-10

**Authors:** Ibraheem M. Rajab, Peter C. Hart, Lawrence A. Potempa

**Affiliations:** Roosevelt University College of Pharmacy, Schaumburg, IL, United States

**Keywords:** CRP - C-reactive protein, MCRP, inflammation, conformational isoforms, blood kinetics, modified/monomeric (mCRP)

## Abstract

C-reactive protein (CRP) is a widely known, hepatically synthesized protein whose blood levels change rapidly and pronouncedly in response to any tissue damaging event associated with an inflammatory response. The synthesis and secretion of CRP is stimulated by interleukin-6, an early pleiotropic cytokine released by macrophages, endothelial, and other cells that are activated when localized normal tissue structures are compromised by trauma or disease. Serum CRP levels can change rapidly and robustly from 10-100-fold within 6–72 h of any tissue damaging event. Elevated blood levels correlate with the onset and extent of both activated inflammation and the acute phase biochemical response to the tissue insult. Because its functional bioactivity as the prototypic acute phase reactant has eluded clear definition for decades, diagnosticians of various conditions and diseases use CRP blood levels as a simple index for ongoing inflammation. In many pathologies, which involves many different tissues, stages of disease, treatments, and responses to treatments, its interpretive diagnostic value requires a deeper understanding of the localized tissue processes and events that contribute signals which regulate protective or pathological host defense bioactivities. This report presents concepts that describe how local tissue activation events can lead to a non-proteolytic, conformational rearrangement of CRP into a unique isoform with distinctive solubility, antigenicity, binding reactivities and bioactivities from that protein widely known and measured in serum. By describing factors that control the expression, tissue localization, half-life and pro-inflammatory amplification activity of this CRP isoform, a unifying explanation for the diagnostic significance of CRP measurement in disease is advanced.

## Introduction

C-reactive protein (CRP) is a key protein of innate immunity. Structurally, it is a non-glycosylated, non-covalently associated protein of five homologous globular subunits arranged in discoid symmetry (1). Each subunit has a calcium-regulated shallow binding pocket for ligands expressing phosphocholine (PC) moieties with all five PC binding pockets expressed on the same face of the flattened pentameric disc. CRP's bioactivities as an effector molecule of innate immunity involves its role as a pattern recognition ligand for exposed PC groups as may be expressed in teichoic acid of Gram positive bacteria or on activated cell membranes where phospholipid bilayers buckle in response to activation signals such as Phospholipase A2 or lipid acyl chain oxidation (2, 3). When bound, CRP is reported to activate endothelial cells, platelets, leukocytes and the complement system, and influence the overall inflammatory response that involves the earliest acute phase host defense response to any inciting threats to tissue homeostasis (4, 5). While CRP has been widely studied as a key protein of the acute phase response (APR), its role in activating and/or regulating host defense processes has remained undefined.

While CRP blood levels change rapidly and pronouncedly with any host defense response to tissue damaging events that triggers inflammation, the lack of understanding of its true function has limited the diagnostic interpretation of its blood levels. On the other hand, assays to quantify CRP in blood have evolved to be rapid, economical and sensitive. Due to its recently availability and accessibility as point-of-care measurements, CRP has replaced the Erythrocyte Sedimentation Rate as the best diagnostic index for ongoing inflammation in an individual. However, CRP blood levels can vary 10-to-100 fold within 6–72 h of the initial tissue insult, its levels are not only used as general identifier of ongoing inflammation but as an index of the extent to which the APR and inflammation are actively stimulated and for how prolonged the inflammation persists. Extreme elevations in blood levels of CRP that persist or recur over time are generally perceived to be a bad omen for disease progression.

One particularly confounding disease in understanding of diagnostic relevance of CRP is cancer. Cancer disease can be variable in that it involves different tissues and pathological stages, can persist for years and can be in progression or remission. Despite these disparate conditions, many publications include discussions of CRP blood levels without elaborating on disease conditions when CRP blood levels were taken. Indeed, reported CRP levels vary from high sensitivity levels (i.e., CRP values <10 μg/ml), to conventional levels (i.e., values above 10 μg/ml), and in certain instances up to more than 200 μg/ml. A consensus understanding of what divergent CRP levels indicate in individual cancer cases as related to both the disease and the progression of disease has been lacking. Exhaustive reviews in the literature (6–9) have revealed that (1) CRP levels above 10 μg/ml are indicative of an ongoing tissue damaging inflammatory response in a patient; (2) CRP levels progressively increasing from 10 μg/ml to more than 100 μg/ml are indicative of progressively more active inflammation and disease progression; (3) CRP levels above 100 μg/ml are prognostic of a poor outcome and/or a failure to respond to therapy; (4) CRP and other inflammatory markers are not useful tools to diagnose the presence of cancer. Because CRP levels increase and decrease rapidly (within days), changing without a memory response, it is important to assess the diagnostic relevance of CRP as a function of when measurements are made, as a function of disease stage, and as a function of patient responses to any treatment. A potential strategy was hypothesized by Coventry et al. (10) to monitor the sequential CRP values in a defined time frame and correlate the CRP levels increase with cancer disease progression; and reciprocal decrease of the sequential CRP values during cancer remission.

This report introduces a unifying explanation for the quantitative and temporal appearance of CRP in blood and its innate bioactivities as the prototypic acute phase reactant. With the newly appreciated understanding that CRP is a dynamic protein that can undergo *in situ* a non-proteolytic conformational change into a unique, distinctive isoform, its role in amplifying and regulating inflammation is described. The protein found and quantified in blood is the pentameric discoid protein (i.e., abbreviated “pCRP” for “pentameric CRP”). When pCRP binds to an activated membrane, biochemical forces contribute to dissociation of the pentamer which induces a pronounced structural rearrangement exposing a cryptic binding site on the dissociated CRP subunits for cholesterol molecules found in lipid rafts. Dissociated CRP subunits are described as “monomeric, modified” CRP (i.e., abbreviated as “mCRP”) (11–16). The mCRP isoform is antigenically distinctive from the pCRP isoform and has significantly reduced aqueous solubility. When mCRP enters into lipid rafts, it triggers intracellular signaling pathways that strongly enhance pro-inflammatory activities generally known to be activated as part of the earliest phases of the acute phase response (17). By carefully evaluating the distinctive bioactivities of pCRP and mCRP, it is now established that pCRP has weak anti-inflammatory bioactivity while mCRP has strong pro-inflammatory bioactivity (4). By introducing and interpreting these novel concepts of the distinctive structural isoforms of CRP, a unified hypothesis for the diagnostic and therapeutic role of CRP is advanced. This report presents concepts of two structural isoforms of CRP with distinctive bioactivities as a relevant index to assess disease progression, and how the natural host defenses respond to disease.

## Historical Reflections on CRP and its Role in Health and Disease

As an evolutionarily conserved protein found in almost all lower species (18), it is tempting to speculate on the fundamental role that CRP may have in regulating innate defenses. Studies have appeared noting that CRP blood levels do increase in children with protein calorie malnutrition (19–21). This observation suggests that even during periods where hepatic gluconeogenesis is activated to supply metabolic fuel, the need for CRP supersedes the need to supply metabolic energy.

Even though the CRP response is associated early during innate immunity, its bioactivities have also been linked to binding immunoglobulin Fc receptors [reviewed in (4)]. Thus, CRP contribute to effector mechanisms with crossover activity between innate and adaptive immunity. However, CRP blood levels fluctuate independently of immunoglobulin concentrations (22) and a CRP response has been measured in agammaglobulinemic patients (23) indicating at least part of its biofunction occurs in the absence of antibody-mediated effector responses.

The extent to which blood levels of CRP increases above 10 μg/ml has been used to differentially diagnose certain diseases (24, 25). For example, CRP levels increase more pronouncedly in rheumatoid arthritis compared to systemic lupus (26). CRP levels are also found higher in bacterial infections than those caused by a virus, being helpful in directing the use of antibiotic therapy (27). CRP levels are also elevated during acute bronchitis compared to episodes of asthma (28). Most fundamentally, the more severe the tissue damage and stimulated inflammatory response, the higher the CRP levels. Higher CRP values are reported to correlate with poorer prognoses in any disease where CRP is measured. By focusing on the relationship of CRP blood levels to tissue damage rather than inflammation, a consensus can be made that CRP levels > 50–100 μg/ml are indicative of tissue damage that is so severe as to threaten survival.

Surprisingly, even though there is a clear association of CRP levels with the inflammatory response that occurs following any tissue damaging event, CRP is not found to selectively localize to injured tissue sites (29). Using intravenously injected ^125^I-labeled CRP in human turnover studies, CRP was found to be a blood protein with a mean plasma half-life of 19 h. Its plasma clearance rate was similar in both normal control and various patient groups including patients with active autoimmune diseases, localized infections and localized cancers. While on the surface the fact that CRP does not locally sequester to the site of injury or disease would appear to be counterintuitive, these results call to question its true relationship as a mediator of host defense processes activated at sites of injured tissues.

## Use of CRP as A Diagnostic Marker in Disease

The US Department of Health and Human Services guidelines for interpreting the diagnostic significance of CRP values defines a difference between “Conventional CRP” values and “High Sensitivity CRP” values (30). FDA guidance is only given for conventional CRP levels, defined as being > 10 μg/ml. While hsCRP levels (i.e., <10 μg/ml) are an area of great research interest, the FDA warns that such values are non-specific and should only be interpreted in combination with a full clinical evaluation. In multicenter studies involving tens of thousands of CRP measurements, the significance of hsCRP levels as having predictive clinical value has been questioned (31, 32).

One explanation for differences in baseline hsCRP levels has been attributed to genetic polymorphisms in the promoter region of the transcribed CRP gene. The gene for CRP is located on Chromosome 1 locus q23.2, the largest human chromosome, having about 8% of total DNA in human cells. Chromosome 1 is reported to have 249 × 10^6^ nucleotide base pairs and > 4,300 genes. The CRP gene is comprised of 2 exons separated by a single intron of 278 nucleotides that includes a dinucleotide GT repeat sequence. Exon 1 encodes for an 18 amino acid leader sequence and the first 2 (of 206) amino acids in the mature CRP subunit and exon 2 codes for the remaining 204 amino acids (33). While genetic polymorphism has been associated with slight changes in circulating CRP levels (34), there is no known significance of such polymorphism on disease risk (35).

In focusing on specific patient ethnicities, genders and general activities rather than disease, differing interpretations and explanations for the diagnostic value of hsCRP have emerged (36, 37). hsCRP levels were found to be higher in women and black ethnicities and were found to decrease in Hispanic men engaged in average to above average physical activity. Of note, vigorous exercise by black and white men appeared to lower hsCRP levels. In this report, no interpretation is given to any CRP level <10 μg/ml (i.e., hsCRP). Emphasis is instead placed on understanding the relationship of conventional CRP levels as an index for and persistence of tissue damage associated with disease and disease progression. The widely accepted association of CRP with inflammation is not challenged. However, since inflammation is a natural response to tissue damage, readers are encouraged to broaden their understanding to associate CRP levels with the presence and extent of tissue damage. In doing so, and by understanding that CRP is not a single, unchanging/rigid structural entity (as outlined below), a common theme for the bioactivities of CRP emerges, defining the understanding of its fundamental role as a key protein of the acute phase host defense response.

## Structural Isoforms of C-Reactive Protein (CRP) Exhibit Distinct Antigenicity and Bioactivity

CRP is widely recognized as a key acute phase reactant found in blood, primarily produced hepatically in response to tissue damage that elicits an inflammatory response (38–44). CRP is a protein of the innate (natural) immune system, providing baseline protection as a pattern recognition molecule, and as a regulator of host defense responses involving tissue barriers, vascular activation, phagocytic responses, and amplification mechanisms. As these host defenses feed into and direct certain responses of the acquired (specific) immune system (3, 45), CRP has been broadly studied as a molecule that can contribute to both positive and negative immune responses to essentially all disease etiologies. While the consequences of an impaired CRP response are unknown, the consequences of an impaired innate immune response include changes in natural resistance to pathogens and the development of autoimmune diseases. While CRP has been recognized for over 70 years as a blood protein whose levels change correlate with ongoing active inflammatory responses, its biological role during such processes remained undefined and controversial with conflicting reports concluding it both promoted and inhibited inflammation (46).

**Figure 1:**
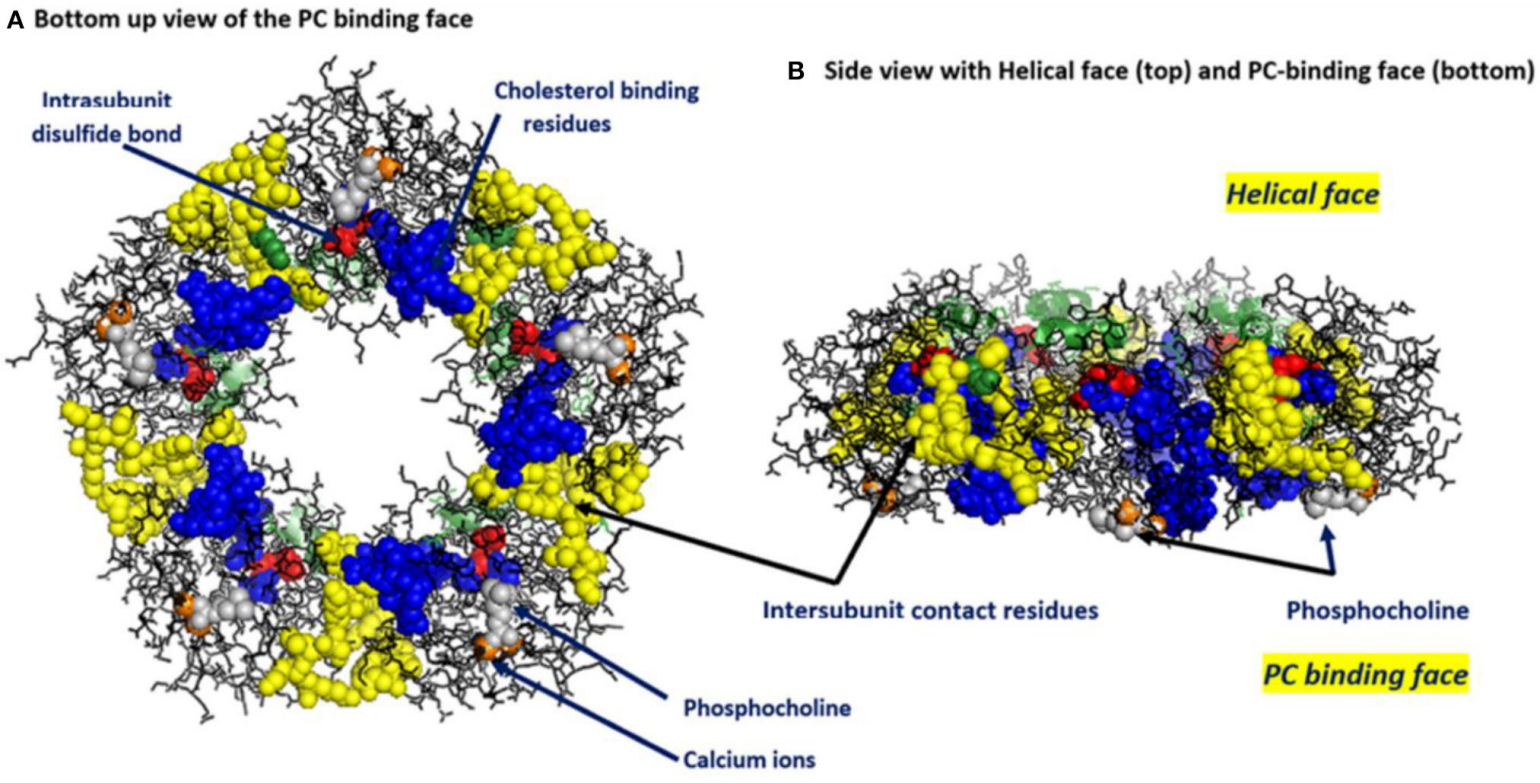
Key structural features of serum-soluble pentameric CRP. **(A)** illustrates the location and orientation of Phosphocholine (PC) binding sites (one per subunit, PC groups shown in gray and involving residues L64, F66, and T76 of each subunit (there are 206 amino acids in each subunit), calcium ions (two per subunit shown in orange, juxtaposed to each PC binding sites and involving residue E147), inter-subunit contact residues (shown in yellow and involving 40YTE42, 115PRVRKSLKK123 AND 197EVFTKP202 and which non-covalently contribute to pCRP pentameric quaternary structure), the one per subunit intrachain disulfide bond (shown in red and covalently linking C36–C97 in each subunit) and the location and orientation of the cholesterol binding residues (shown in blue and involving 35VCLHFYTELSSTR47) in relation to the PC-binding sites and the inter-subunit contact residues (PDB code: 1B09) [residues identified in Shrive et al. (1)]. **(B)** illustrates the orientation of these same residues when the discoid protein is laid flat (i.e., side view). This view illustrates that all PC binding sites are on one face of the flattened discoid structure and shows how a single helical secondary structure (involving residues 168PDEINTIYL176) is oriented on the top of the opposite face of the discoid protein.

CRP is a highly soluble protein comprised of five non-glycosylated globular subunits arranged in cyclic symmetry (Figure 1). Its three-dimensional structural conformation has been resolved using X-ray crystallographic analyses (47, 48), as well as by deduction from the X-ray crystallographic analysis of the Serum Amyloid P component (SAP), which shares 51% amino acid sequence homology with CRP and 59% nucleotide sequence identity (49, 50).

CRP levels in blood change markedly within hours to days of any event that involves tissue damaging pathology. While some variability exists in laboratory methods used to establish baseline levels, healthy individuals are found to have blood levels between 1 and 3 μg/ml (25). In response to tissue trauma, blood levels increase within 6–48 h to levels generally related to the extent of the tissue damage. As inflammation is a natural host defense response to tissue damage, CRP levels have historically been diagnostically associated with inflammation rather than tissue damage. More directly, CRP levels change in response to the presence and extent of tissue damage which in turn activates the acute phase inflammatory response to the injury. Acute inflammation, occurring within minutes to hours of the tissue damaging event, is generally regarded as beneficial to host defense response. Chronic inflammation, which can persist for days, weeks, months, or years, can lead to healthy tissue damage and disruption, weakened host defenses, and problematic pain responses. This distinction is of relevance to a discussion of CRP because CRP is now known to exist in at least two isomeric structural forms with opposing activities on the inflammatory response (see below). The CRP isoform recognized and measured in blood is the highly soluble, non-covalently associated cyclic pentamer (described as pentameric or “pCRP”). pCRP has recently been shown to have weak anti-inflammatory bioactivities. When the pentamer is coerced to dissociate into individual subunits, however, it undergoes a non-proteolytic, substantial conformational change into a structurally, antigenically and biologically distinctive molecule described as modified, monomeric CRP (i.e., “mCRP”). mCRP is a short-lived isoform with potent pro-inflammatory, acute phase amplification bioactivities that are associated with the earliest phases of the acute, beneficial inflammatory response (4).

**Figure 2:**
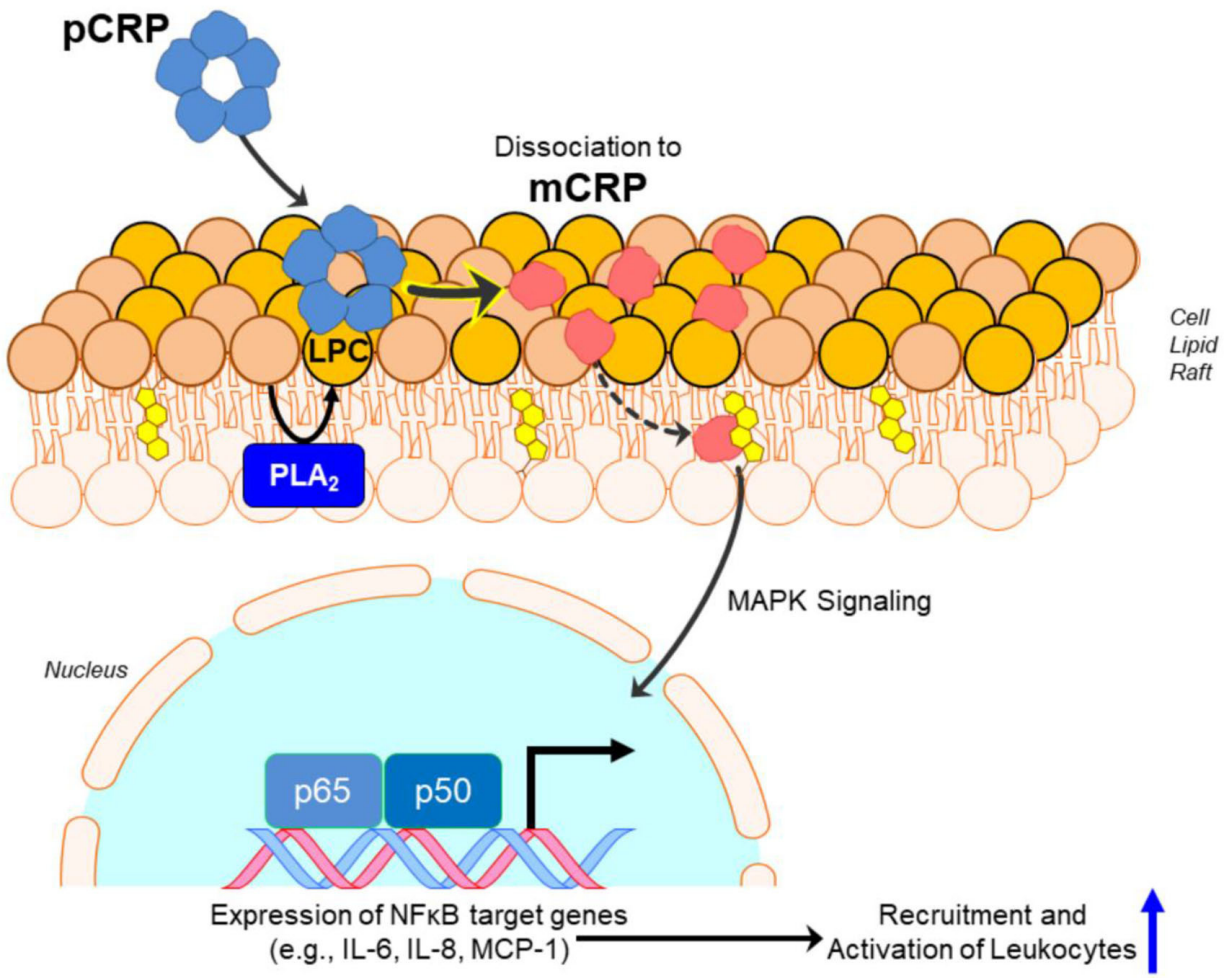
Conversion of pCRP to mCRP induces inflammatory signaling. Monoacyl phosphatidylcholine (aka Lyso-PC or LPC) generated by phospholipase A_2_ (PLA_2_) in the lipid bilayer, or by oxidation of lipid acyl chains by reactive oxygen species, promotes the binding and dissociation of pentameric CRP (pCRP) to monomeric CRP (mCRP). Formation of mCRP exposes a cholesterol binding sequence such that mCRP enters cholesterol-rich lipid rafts, activating intracellular signaling pathways involving NF-κβ-regulated translation of proteins involved in pro-inflammatory responses.

pCRP is a substrate for the formation of mCRP. At local sites of tissue damage, pCRP can be induced to convert into mCRP by binding to and interacting with membrane lipids [initially using its calcium dependent binding specificity for ligands expressing phosphocholine (PC) ] (51). Membrane PC groups are most accessible for pCRP binding after Phospholipase A2 cleaves an acyl chain from a phospholipid, creating the detergent-like lipid, monoacyl (Lyso)-PC. Membrane bound pCRP is brought into juxtaposition with apolar regions of the membrane, which contributes biochemical energies needed to dissociate the pentamer. Furthermore, the structural change of each CRP subunit that accompanies membrane interaction, exposes a new binding site (only expressed on the mCRP isoform) for cholesterol found in lipid rafts (2, 17, 52) (Figure 2), membrane microdomains that regulate cellular signaling pathways of importance in health and disease (53).

The expression of mCRP must be tightly regulated and controlled; its half-life must be short and localized. As pCRP is a substrate for the formation of mCRP, the relative level of pCRP measured in blood will depend in part on the rate at which pCRP is converted into mCRP. During the earliest minutes of tissue trauma, the conversion of pCRP into mCRP is efficient and rapid. Expressed mCRP, which has potent proinflammatory bioactivities, then upregulates and amplifies the acute phase of the inflammatory response. Over the next minutes to hours as the immediate threat to tissue homeostasis is controlled, the conversion of pCRP to mCRP slows, leading to an increased measurement of pCRP in blood. Only the pCRP antigen is measured and interpreted as a diagnostic maker in blood. As the mCRP antigen forms and quickly sequesters into membranes and tissues involved in the inflammatory response, aqueous solubility is reduced and antigenicity is masked, making it a difficult antigen to detect in body fluids. As the pCRP isoform has weak anti-inflammatory activities while the mCRP isoform has strong pro-inflammatory activities, the level of pCRP that is soluble and easily quantified in blood reflects on the extent of a weakened inflammatory, unamplified acute phase response. As pCRP hepatic synthesis is regulated by cytokines released by damaged endothelial cells (38, 54), persistently elevated synthesis and plasma levels of CRP is more likely directly related to tissue damaging pathologies rather than the inflammation. Chronic (unamplified) inflammation could persist in such a situation, exacerbating tissue destructive processes and impairing wound healing and repair to reestablish health homeostasis.

Taken together, pCRP levels that remain elevated at and above values generally considered to be diagnostic of ongoing inflammatory responses reflect not on the presence of generalized inflammation, but rather the degree to which acute, amplified (beneficial) inflammation is stimulated. Higher pCRP levels can be an index for inefficient or inhibited processes by which pCRP converts into mCRP. If mCRP is not formed, inflammation cannot be amplified to its acute phase, resulting in a persistent chronic response and pathological complications.

## Kinetics of the CRP Response

Plasma CRP is synthesized and released by hepatocytes in response to IL-6, IL1β, IL-17 and stress signals that accompany vascular stimulation associated with tissue damage (54, 55). Prior to receiving stress signals, hepatocytes will slowly release basal levels of CRP that were pre-synthesized and stored in intracellular vesicles. The secretory rate of CRP increases 6-fold in response to stress signals (43), which also initiates new protein synthesis. In healthy individuals, the normal synthesis rate is 1.5 μg/kg-hr.; in diseased states, its synthesis rate in reported to range from 43.3 to 103.4 μg/kg-h (i.e., a 30-70-fold increase). These values calculate such that an average person will synthesize 2.4 mg of CRP/day, increasing up to 174 mg/day in response to an exacerbating event (29). CRP's fractional catabolic rate (i.e., its rate of consumption) is reported to be independent of its plasma concentration indicating changes in CRP blood levels during an acute inflammatory response reflect on an increased synthesis rate and not on an increased rate at which it is utilized. These observations appear to confound the understanding of CRP as the prototypic acute phase reactant which would be expected to be involved with and consumed by inflammatory processes.

**Figure 3:**
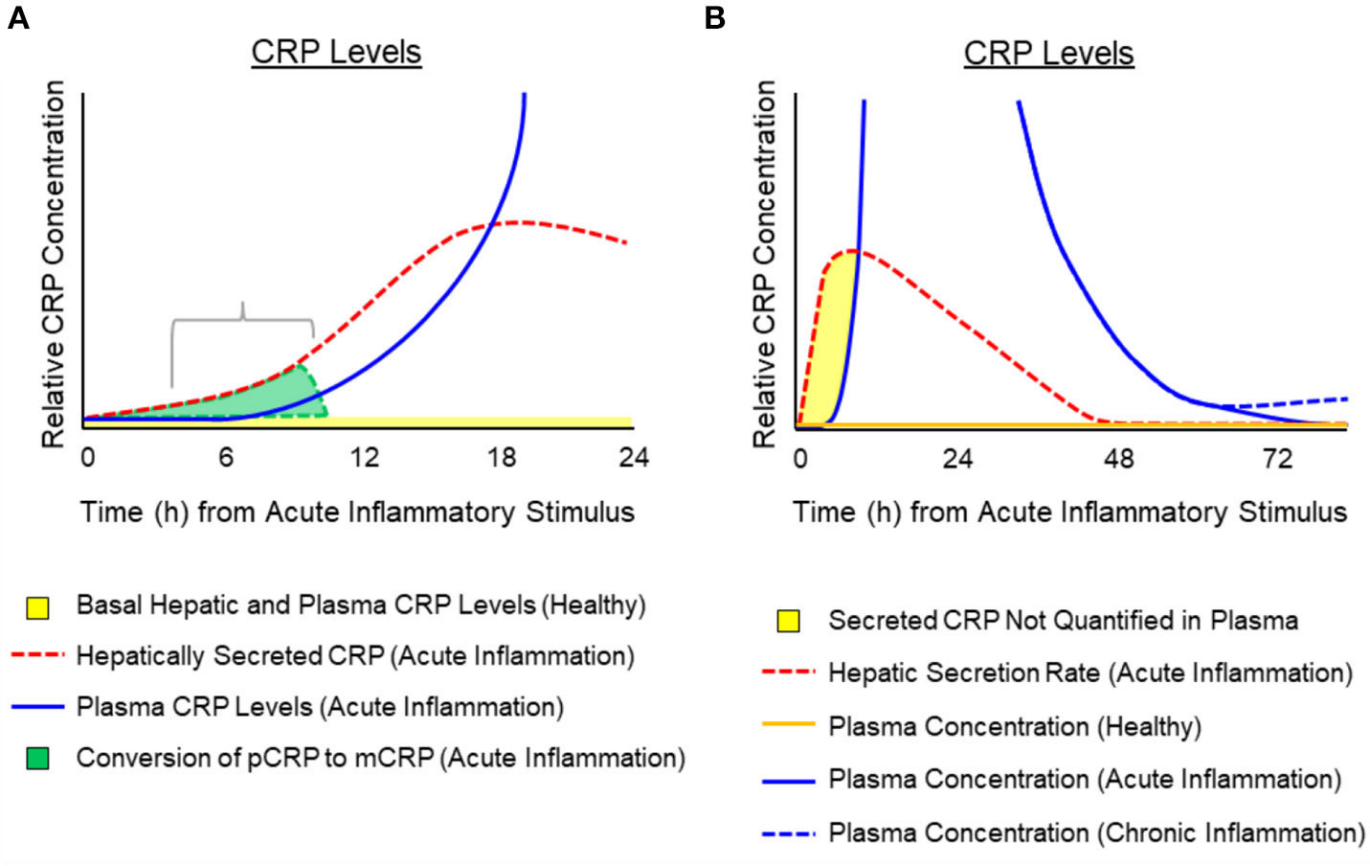
Temporal depiction of the hypothesized conversion of pCRP into mCRP and the appearance of pCRP in blood after activation or an acute phase response. Schematic representation for the relative amounts of pCRP released from hepatocytes in response to cytokine signals (e.g., IL-6) and its appearance in the blood. **(A)** Depicts that while pCRP is hepatically secreted within minutes of acute phase response signaling (dotted red line) measured plasma levels of CRP display a lag (i.e., 6–12 h) (blue solid line). Hepatically released CRP can be from both pre-synthesized CRP stored in vesicles, or from *de novo* synthesis. In the first 12 h. of an inciting stimulus, the pCRP that is released from hepatocytes, but which is not quantified in blood is converted at the site of tissue damage into mCRP (gray shaded area). **(B)** Depicts a generalized time course for the hepatic secretion of pCRP (red dotted line), the relative amount of pCRP measured in blood (solid blue line as may occur during acute inflammation associated with major tissue damage; dotted blue line as may occur with low grade, chronic inflammation with lesser tissue damage). Plasma concentration of pCRP in healthy individuals (i.e., baseline CRP levels of < 10 μg/ml) is shown as a solid orange line. The shaded yellow area depicts a time period in which pCRP is secreted but rapidly consumed so that it is not quantified in plasma. The consumption of pCRP into mCRP would involve membrane binding to activated cells (e.g., endothelial cells, platelets, leukocytes) and entry into lipid rafts where mCRP activates signaling pathways to stimulate immediate, amplified pro-inflammatory responses to the threat.

While synthesis and hepatic release rates of CRP in response to cytokine stimulation have been reported (29, 41, 43, 56), measured increases in CRP blood level appear to involve a lag of 6–12 h after stimulation (40, 57, 58). Its level will continue to increase between 6 and 72 h as a result of both increased synthesis and release from hepatocytes. The exact kinetics of CRP appearance in blood during the first minutes to hours of an acute phase stimulation requires additional, focused experimentation. In reflecting on published quantification kinetics and the immediate, 6-fold increase in CRP secretion rate during the first moments of a stimulating cause, and by taking into account the propensity for CRP to dissociate into an isoform with distinctive structural, solubility and antigenicity differences from that CRP molecule measured in blood (4, 17, 59–63), the apparent lag phase was interpreted to represent a physiologically relevant finding in the bioactivity of CRP. If CRP is released from hepatocytes within minutes of acute phase stimulation but is not detected in blood for hours, such data are consistent with a rapid conversion of soluble, pentameric CRP into its distinctive structural and antigenic mCRP isoform during these early moments. Indeed, at the site of tissue damage, as reactions of acute inflammation are activated, dissociation of pentameric CRP into conformationally unique mCRP would represent an important regulatory signal that functionally contributes a potent pro-inflammatory amplification signal to immediately and aggressively respond to the threat to tissue integrity. As the need to maintain acute inflammation remains, pCRP localizes and converts to mCRP to maintain and amplify the acute phase response. When amplified, acute inflammation subsides and the rate of pCRP conversion to mCRP slows, pCRP is no longer consumed, which results in increased levels appearing in blood (i.e., after a 6–12-h lag). These concepts involving kinetic changes of hepatic secretion of pCRP during early and late acute inflammation, and the hypothetical impact of the timing and rate of conversion of pCRP to mCRP are illustrated in Figure 3. This illustration depicts the potential relationship between CRP production and conversion into the mCRP isoform during the early phases of acute inflammation, while accounting for the latent increase in significantly elevated levels of pCRP in blood. Conformational activation is a basic tenet of biochemical signaling control (64, 65). Herein, we discuss CRP as a protein requiring conformational changes to elicit and control key host defense bioactivities. Furthermore, the widely held notion that CRP is the prototypic acute phase reactant needs to be refined to more specifically associate the mCRP isoform (rather than the pCRP isoform measured in blood) as the true pro-inflammatory “acute” reactant.

## The Pro- or Anti-Inflammatory Activity of CRP Is Defined by Its Conformation

Over many decades, CRP has been studied as both a contributor to and protector from the pathologies associated with disease. A definitive activity remained elusive for many years as conflicting reports described CRP as both pro- and anti-inflammatory activities, and as both pro- and anti-thrombotic [reviewed in (4)]. Because of this mechanistic uncertainty, CRP could only be described and used as a diagnostic marker of clinical situations involving some level of inflammation with no true understanding of its mechanism (s) of action. Because its blood levels increase and decrease rapidly over days it has only been of limited use in helping devise treatment strategies or in understanding disease pathologies.

The confusion concerning CRP's bioactivities only recently began to clarify when it was recognized that the serum soluble non-covalently associated pentameric CRP structure could be induced to dissociate into monomeric subunits. When separated, the biochemical energies involved in the folding and packaging of each subunit both individually and as a non-covalently associated pentameric multiyear, rapidly and irreversibly redistribute such that the subunits modify into a unique, non-proteolyzed isoform which is described as modified, monomeric CRP (i.e., mCRP). mCRP can be expressed from pCRP under physiological conditions that occur at sites of where endothelial cells are activated. At such sites, pCRP will bind as a function of calcium to its primary ligand – phosphocholine (PC), which only become more exposed when diacyl phosphatidylcholine lipids are hydrolyzed by phospholipase enzymes, producing monoacyl (Lyso) phosphatidyl choline (i.e., Lyso-PC) (2). PC bound pCRP is brought into close proximity to surface membrane charges and to apolar lipid regions which contribute the biochemical energies needed to dissociate pCRP subunits which then spontaneously rearrange to express mCRP structure and its distinctive immunological and biochemical attributes (Figure 2).

**Figure 4:**
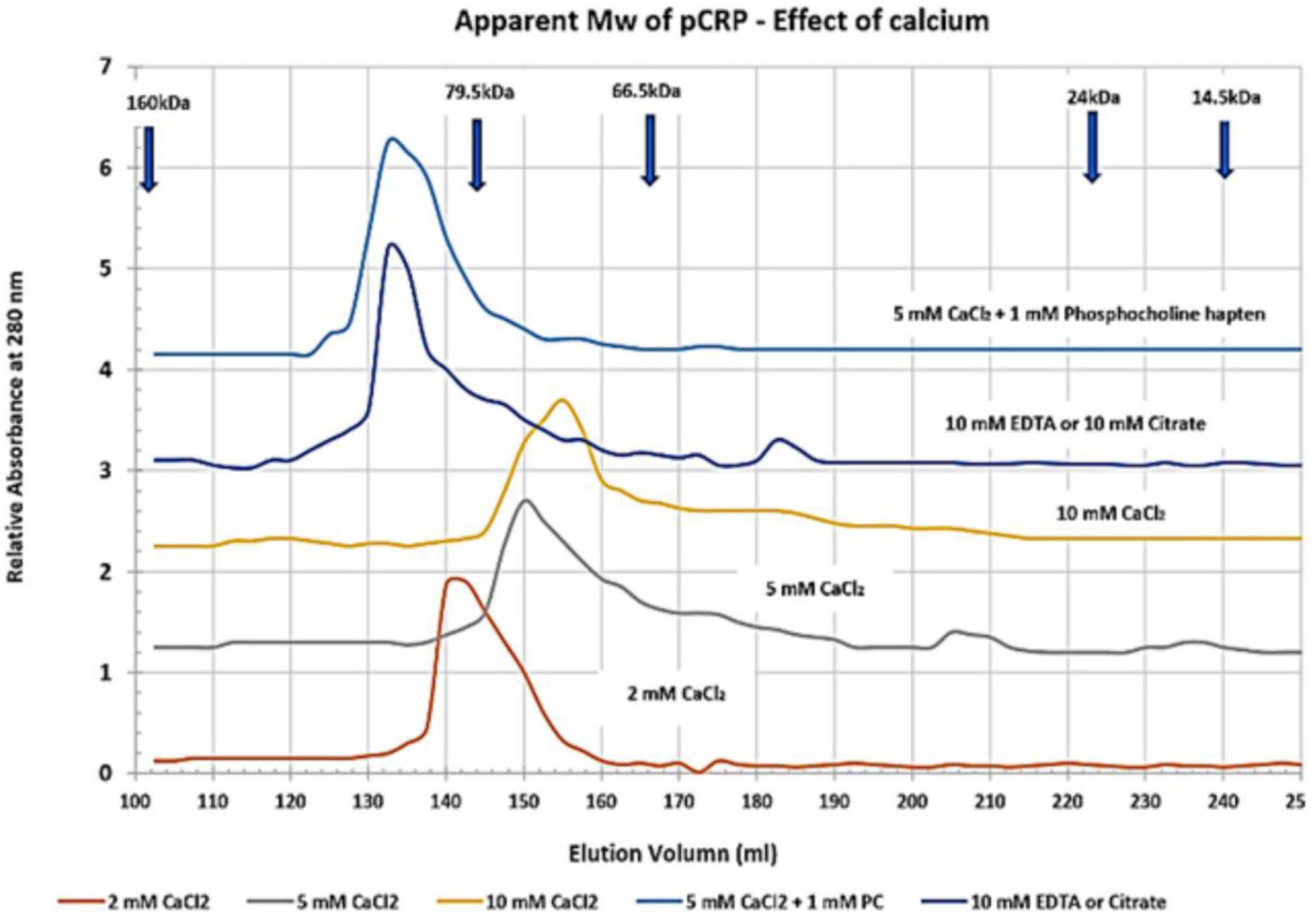
Effect of calcium on the apparent molecular weight of pentameric CRP. Sephadex 200 dextran gel filtration chromatography resin was equilibrated in 25 mM Tris-HCl, 0.15 M NaCl buffer (pH 7.4) (TBS) containing 2, 5, or 10 mM Calcium chloride (TBS-Ca), 10 mM EDTA (TBS-EDTA), or 10 mM Citrate (TBS-citrate). Purified pCRP (1 mg) was pre-equilibrated in each column buffer and was chromatographed. Control proteins including IgG (Mw 160,000), Transferrin (Mw 79,500), BSA (Mw 66,500), Chymotrypsin (Mw 24,000) and Lysozyme (Mw 14, 300) were similarly pre-equilibrated and chromatographed. The elution profile for CRP was also assessed using 5 mM CaCl_2_ containing 1 mM Phosphocholine hapten (PC) as a specific ligand known to bind each subunit of pCRP as a function of calcium. All standard proteins chromatographed with the same elution volume in each of the buffers studied. The correlation coefficient (*R*^2^) of the Mw Vs elution volume standard curve was 0.9766. The apparent Mw of pCRP in 2 mM CaCl_2_ was 115,093 (2 mM CaCl2 is a physiological concentration of calcium; this Mw agrees closely with the calculated Mw of pCRP based on its amino acid sequence). The apparent Mw of pCRP reduced to 96,828 in 5 mM CaCl_2_ and 86,160 in 10 mM CaCl_2_. When either calcium chelator was used, the apparent Mw of pCRP increased to 133, 122. When PC hapten was bound to pCRP in 5 mM CaCl_2_ (5 PC sites/pCRP), the pCRP did not appear to contract but assumed a Stoke's radius similar to that observed in when calcium was chelated. These data indicate pCRP will compact or expand from its calculated Mw (115,235) (https://www.ncbi.nlm.nih.gov/protein/NP_001315986.1?report=fasta&from=19, accessed on March 30^th^, 2020) as a function of calcium and/or or chelation, and as a function of ligand binding.

By recognizing that CRP can exist as more than a stagnant, unchanging cyclic pentameric discoid protein, but also as a dynamic structure that can be induced to undergo conformational activation, a reason for its appearance and pharmacokinetic changes in blood levels, and its bioactivity during an inflammatory acute phase response begins to unfold. The concept of conformational activation of proteins is a widely accepted for many biochemical systems including allosteric signaling (66), enzyme catalysis (67) and ion-gated channel activities (68). CRP, as a tightly packed non-covalently associated pentameric protein with identical non-glycosylated globular subunits, would be a strong candidate for such an activation mechanism as it will express five stimulatory proteins from one serum-delivered substrate that forms on activated endothelial tissues. The tight packing of pCRP involves the binding of two calcium ions per subunit (1). Calcium has direct effect on CRP packing as summarized in Figure 4, which shows how the apparent size of pCRP (i.e., its Stoke's radius) changes as a function of the amount of calcium included in CRP solutions. In 2 mM calcium chloride, a physiological concentration of calcium in blood, pCRP migrates with an apparent molecular weight of 115 kDa – in close agreement with its calculated molecular weight based on its primary protein sequence. As calcium is increased to 5 and 10 mM, however, pCRP appears as a smaller (more compacted) protein (smaller Stoke's radius). In the tightly packed configuration, pCRP has an electron microscopy average diameter of 10.42 ± 0.08 nm (69) and is resistant to proteolysis (70). When calcium is chelated using citrate or EDTA in chromatographic buffers, pCRP appears to “swell,” having an apparent molecular weight of 125–140 kD (i.e., a larger Stoke's radius). In early studies establishing isolation protocols for CRP from blood, many chromatographic steps used 0.05 M citrate buffers (71). CRP's larger apparent size led the authors to conclude that at least some CRP could exist as a hexameric protein.

Calcium has long been known to regulate CRP binding reactivity for PC; the calcium binding sites of each CRP subunit frame its PC binding pocket (see Figure 1). These data show calcium can also affect pCRP packing and proteolytic susceptibility, and suggest CRP has a capacity to change its three-dimensional protein structural packing (i.e., its conformation). Swanson et al. (72) reported that calcium could affect CRP structure in a way to regulate binding of CRP-specific monoclonal antibodies. Furthermore, the physiological relevance of structural changes in CRP was demonstrated in electron microscopy and adsorption studies of pCRP onto Lyso-PC containing monolayers. Binding pCRP to the PC-containing lipid monolayers required calcium and led to a two-stage alteration in the pentameric CRP structure. Initially, membrane bound pCRP changed conformation into a hybrid molecule called membrane-associated-mCRP (or mCRP_m_). With lengthened incubation times, the second alteration occurred, completely converting the protein into its mCRP isoform (17, 73). Additional studies have reported on calcium-dependent pCRP binding to Lyso-PC activated platelets and neutrophils, producing a structurally altered CRP hybrid molecule described as pCRP^*^ (5, 12). In each instance, potent pro-inflammatory bioactivities of CRP are expressed correlate with the structural changes of pCRP into mCRP.

The mCRP isoform has significantly different solubility and antigenicity compared to the pCRP isoform. mCRP enters and associates with membrane lipids, into cholesterol rich lipid rafts (52, 73) and is not freely soluble in aqueous phase, only being found in body fluids associated with micro-vesicles that are sloughed off activated endothelial cells as part of the activated inflammatory response (74, 75). Monoclonal antibody reagents were developed to clearly define and differentiate the pCRP antigen from the mCRP antigen (61, 62). Furthermore, by carefully identifying conditions which contribute to the conversion of pCRP into mCRP, and by using reagents that certifiably distinctive as predominantly pCRP and mCRP, key physiological distinctions between each isoform were identified.

While the pCRP antigen is mainly found in blood, it can occasionally be found weakly associated with vascular surfaces and easily eluted with washing. The mCRP antigen, in contrast, is found as a naturally occurring molecule within a wide variety of normal tissues, in particular at the intima, media and adventitia of healthy blood vessels and in fibrous tissues of the skin (76–78).

While the pCRP antigen is resistant to proteolysis, the mCRP antigen can be proteolyzed by a variety of neutrophil-derived peptidases (79–81). Peptides derived from the proteolysis of mCRP have been shown to inhibit activation of platelets and neutrophils (82), downregulating the potent pro-inflammatory activities of the intact mCRP protein. Hence, there is a direct feedback mechanism elicited by neutrophil-derived enzymatic proteolysis that can quickly reverse the pro-inflammatory bioactivity of mCRP.

For decades, CRP has been appreciated solely as a diagnostic marker for ongoing inflammation. With the now recognized appreciation that CRP is a dynamic protein that can undergo a pronounced structural and functional change, and by understanding the conditions that contribute to such change *in situ*, insights can now also be made into the therapeutic relevance of CRP in health and disease. Notably, pCRP will change into mCRP by interacting with activated membranes (83) using a process that involves an intermediate form of CRP described as mCRP_m_ (17) or pCRP^*^ (63). The intermediate structure represents initial stages of subunit dissociation in which the still pentameric protein begins expressing antigenic and functional attributes now known to be characteristic of mCRP. As the pentamer fully dissociates into its monomeric form (i.e., mCRP), it inserts into cholesterol rich membranes (lipid rafts) and supplies an activation signal for pro-inflammatory pathways associated with the acute phase inflammatory response. Activated membranes are known to slough microvesicles (aka microparticles), which have been reported to contain mCRP (63, 74, 75, 84, 85). To date, while a reliable, direct, quantitative blood-based assay for mCRP remains elusive, when developed, it will provide a new perspective on the role CRP has as a regulator of inflammation, not only as a diagnostic marker but as a therapeutic parameter in deciding disease conditions and treatment options.

By influencing or controlling the conversion of pCRP to mCRP, it may be possible to therapeutically control the rate and extent of anti- and pro-inflammatory responses. Indeed, Caprio et al. (2), have discussed using inhibitors of Phospholipase A_2_ (PLA_2_) to mitigate the production of lyso-PC which is a critical step in binding pCRP and bringing it into juxtaposition to the apolar membrane zone (83). Pepys et al. (86) designed a small molecule bi-valent compound having phosphocholine groups on opposite ends of a linking group. As PC is the primary ligand for CRP, and since the PC binding sites are all the same face of the pentameric structure (see Figure 1), this bis-PC compound would link to two pentamers face to face, essentially forming a CRP decamer. By occupying 10 PC sites, and by complexing 2 CRP pentamers, CRP would be unable to bind membrane associated PC and interact with apolar membrane regions that contribute biochemical energies needed to conformationally change pCRP into mCRP. Braig et al. (63) described how this bis-PC compound influenced CRP, pCRP^*^ and mCRP reactivities with microvesicles. All these examples offer evidence of some strategies being studied and developed to control inflammation by controlling, not the quantity of CRP in blood, but in the way and extent that pCRP converts into mCRP.

## Summary and Conclusion

Readers are referred to the following manuscripts that detail the expression of mCRP from pCRP and describe its potent pro-inflammatory bioactivities (4, 5, 11, 12, 63, 73, 87–91). The focus of this report is to provide a new paradigm to understanding what diagnostic CRP levels indicate and in introducing the connection between CRP and inflammation-associated tissue damage. By understanding that the pCRP protein is fundamentally a serum soluble, precursor protein with significant stored potential energies, its diagnostic utility begins to take on a new meaning. The potential energy needed to stimulate and amplify acute inflammation gets released by the spontaneous conformational change that occurs simply when the pentamer is dissociated. The mCRP isoform of CRP is, in fact, the “prototypic acute phase reactant.” Its proinflammatory activities are potent, localized and short lived. Any pCRP that is present during the earliest phases of the host defense response to tissue damage, will be readily converted to mCRP. When expression of mCRP slows or stops, or when mCRP is destroyed by proteases, acute inflammation slows down to chronic inflammation. pCRP, which is synthesized and secreted by hepatocytes is the substrate for the formation of mCRP. It will only start to accumulate in blood after the conversion to mCRP slows down (e.g., after 6–10 h. of the inciting cause). Measured elevation in blood pCRP is more diagnostic of a chronic inflammatory response, which, if prolonged or severe enough, will lead to exacerbated tissue damage caused by prolonged neutrophil stimulated, non-specific effector responses involving secretion of reactive oxygen species and hydrolytic enzymes into affected areas.

## Author Contributions

IR, PH, and LP equally contributed to the concepts of writing of and editing of this manuscript. LP developed concepts, reagents to study isoforms, and especially relevant to the mCRP isoform. PH contributed to inflammation concepts and produced figures presented. IR contributed extensively to summarizing literature reports including data. All authors contributed to the article and approved the submitted version.

## Conflict of Interest

The authors declare that the research was conducted in the absence of any commercial or financial relationships that could be construed as a potential conflict of interest. The reviewer KP declared a past co-authorship with one of the authors LP to the handling editor.
